# Identification of Metabolism-Related Hub Genes in Heart Failure via Comprehensive Transcriptome Analysis

**DOI:** 10.3390/genes16030305

**Published:** 2025-03-03

**Authors:** Hanlin Peng, Boyang Lv, Junbao Du, Yaqian Huang, Qinghua Cui, Chunmei Cui, Hongfang Jin

**Affiliations:** 1 Department of Pediatrics, Peking University First Hospital, Beijing 100034, China; 2 State Key Laboratory of Vascular Homeostasis and Remodeling, Peking University, Beijing 100191, China; 3 Department of Biomedical Informatics, School of Basic Medical Sciences, Peking University, Beijing 100191, China; 4 School of Sports Medicine, Wuhan Sports University, No. 461 Luoyu Rd., Wuchang District, Wuhan 430079, China

**Keywords:** heart failure, metabolism, transcriptome analysis

## Abstract

Background: Metabolic dysfunction is a key driver of heart failure (HF) progression. Identifying metabolic hub genes in HF may reveal novel therapeutic targets. Methods: Transcriptomic datasets from HF patients (GEO database) and metabolism-related genes (PathCards) were analyzed. Differentially expressed genes (DEGs) were intersected with metabolism-related genes, followed by the application of the LASSO, Random Forest, and XGBoost algorithms to prioritize hub genes. Candidate genes were validated via WGCNA, an HF mouse model, and plasma metabolomics. Diagnostic performance and metabolic associations were assessed using ROC analysis and ssGSEA. Results: We identified 1115 HF-associated DEGs (701 upregulated, 414 downregulated), with 119 linked to metabolism. The machine learning algorithms prioritized five genes, including *SDC2*, which was also validated using WGCNA and the mouse HF model. *SDC2* mRNA and protein expression levels were markedly elevated in HF and demonstrated strong diagnostic accuracy. ssGSEA revealed the expression of *SDC2* was correlated with dysregulated metabolic pathways, including fatty acid biosynthesis and glycerolipid metabolism, which are potentially associated with metabolic alterations in HF. Conclusions: SDC2 emerges as a central regulator bridging metabolic dysfunction and HF pathogenesis, showing potential as a diagnostic biomarker and therapeutic target.

## 1. Introduction

Heart failure (HF) is a prevalent chronic cardiovascular disease, affecting about 1% to 2% of the adult population worldwide, with an annual mortality rate of about 10%. It ranks among the foremost causes of death globally [1,2,3]. Mortality due to HF within five years is as high as 50%, which is comparable to some malignancies [4]. Despite the rapid development of treatment strategies, the prevalence of HF is still increasing worldwide due to its complex pathogenesis, increasing aging population, and poor control of risk factors, including hypertension, diabetes, and obesity [5,6]. It is crucial to understand the pathogenesis of HF and find novel targets for its treatment.

Since cardiac contraction continually requires substantial quantities of ATP, cardiac metabolism is essential for cardiac function, and cardiac metabolic remodeling is recognized as an important therapeutic target in HF [7]. Reduced mitochondrial oxidative capacity, inadequate coupling of glycolysis and glycoxidation, and changes in fatty acid oxidation all lead to an energy deficit in the failing heart, which in turn causes structural and functional changes in the heart [8]. In heart failure, metabolic alterations include impaired fatty acid oxidation, increased glucose metabolism, and the accumulation of toxic lipid metabolites such as triglycerides, diacylglycerols, and ceramides, which contribute to mitochondrial and cardiac dysfunction [9,10]. Moreover, the metabolites generated by the remodeling metabolic network can also directly regulate signaling cascades, protein function, gene transcription, and epigenetic modifications [11]. In addition, metabolic changes in non-myocardial cells, including fibroblasts, endothelial cells, and immune cells, also contribute to the development of cardiac pathology [11]. Increasing evidence shows that improving metabolic remodeling in heart failure can play a role in the prevention and treatment of HF. Therefore, exploring the hub genes regulating metabolic remodeling in HF can help identify and control the progression of HF.

The rapid development of high-throughput detection methods such as RNA sequencing has resulted in massive biomedical data, providing strong support for the in-depth exploration of the pathogenesis of diseases. Bioinformatics methods for further mining and reanalysis of these data can be used to identify potential biomarkers. Subsequent validation using methods such as cross-validation, experimental validation, and clinical samples have significantly improved the efficiency and accuracy of screening. This research approach has become one of the classical methods for studying the pathogenesis of diseases and screening biomarkers. Therefore, this study aimed to identify hub genes related to metabolism in HF through comprehensive bioinformatics analyses and then explore their potential mechanisms in the progression of HF so as to offer novel targets and ideas for the mechanisms, diagnosis, and therapy of HF.

## 2. Materials and Methods

### 2.1. Data Preprocessing and Integration

**Figure 1:**
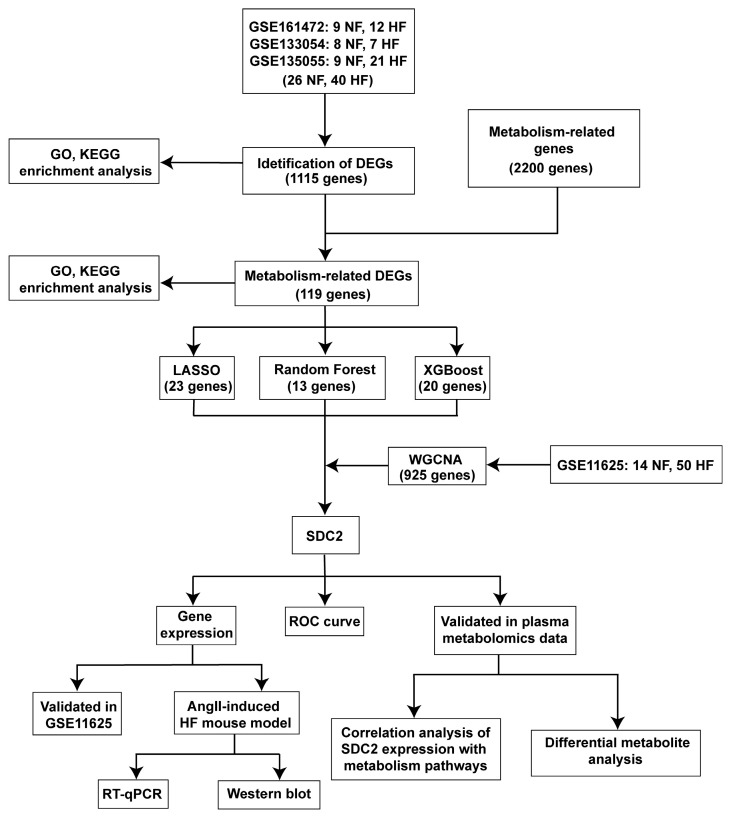
Flow chart.

Transcriptome datasets related to HF were obtained from the GEO database (https://www.ncbi.nlm.nih.gov/geo/ (accessed on 17 December 2024)). The selection criteria were as follows: expression profiling via RNA-seq, samples from Homo sapiens, and cardiac tissue specimens. Finally, the GSE161472 [12], GSE133054 [13], GSE135055 [14], and GSE116250 [15] datasets were included in this study. The comprehensive comparison of the four datasets is provided in Table 1. To enhance the robustness and reliability of the analysis, three datasets (GSE161472, GSE133054, and GSE135055) were integrated in R using the “sva” package (version 3.54.0), and batch effects were removed for subsequent differential gene analysis and enrichment analysis. The GSE116250 dataset was used for WGCNA. Finally, 2200 genes associated with metabolism were downloaded from the PathCards database (https://pathcards.genecards.org/ (accessed on 17 December 2024)). The flow chart of this study is presented in Figure 1.

### 2.2. Identification of Differentially Expressed Genes (DEGs) in HF and Related Enrichment Analysis

Differential expression analysis between different groups was conducted using the “DESeq2” package (version 1.46.0) with the criteria set to Foldchange > 1.5 or Foldchange < 0.67 and adjusted *p*_value < 0.05. The DEG expression data were visualized in a clustered heatmap and volcano plot using the “pheatmap” (version 1.0.12) and “ggplot2” (version 3.5.1) packages. Metabolism-related DEGs were generated by intersecting DEGs and metabolism-related genes. Pathway enrichment analysis for Gene Ontology (GO) was conducted using the enrichGO function in the “clusterProfiler” package (version 4.14.4), and Kyoto Encyclopedia of Genes and Genomes (KEGG) enrichment analysis was conducted using the enrichKEGG function in the “clusterProfiler” package.

### 2.3. Machine Learning

Machine learning algorithms, including LASSO (least absolute shrinkage and selection operator) regression [16], Random Forest (RF) [17], and XGBoost (eXtreme Gradient Boosting) [18], were used for variable screening. Lasso regression enables the selection of variables by incorporating an L1 regularization term into the loss function (the sum of the absolute values of the variable coefficients) to compress the model so that some coefficients are reduced to zero. Lasso can effectively deal with the multicollinearity of parameters, control the complexity of the model, and avoid overfitting. LASSO regression was conducted using the “glmnet” package (version 4.1.8) to identify the optimal regularization parameter (lambda). The Random Forest algorithm is an ensemble learning algorithm consisting of multiple decision trees that perform classification and regression. The variable importance score is obtained according to the variable’s contribution to the final prediction result. The “randomForest” package (version 4.7.1.2) was used to perform Random Forest machine learning. XGBoost is an efficient, flexible, and widely used machine learning algorithm based on the Gradient Boosting framework, which aims to find the optimal prediction model after multiple iterations. XGBoost automatically evaluates the importance of each variable during the training (the importance is usually based on the contribution of the variable to the model’s performance improvement when building the decision tree) and then manually filters the key variables. The “xgboost” package (version 1.7.8.1) was used to perform XGBoost machine learning.

### 2.4. Weighted Gene Co-Expression Network Analysis (WGCNA)

WGCNA is a systems biology approach mainly used to discover gene modules that show co-expression patterns [19] and to correlate these modules with sample characteristics (such as clinical features and phenotypes) to identify key driver genes or biomarkers. This analysis was performed using the “WGCNA” package. The specific steps are as follows: import the standardized data to create a scale-free topological network; select the soft threshold index (β) that meets a fit index greater than 0.85 through the function pickSoftThreshold to ensure that the topological structure of the network has a good fitting degree; construct the adjacency matrix and topological overlap matrix (TOM): use the function adjacency to calculate the adjacency matrix between genes, and then convert the adjacency matrix to a TOM through the TOMsimilarity function; identify gene modules by using unsupervised clustering methods, and perform cluster analysis based on the hierarchical clustering results using function hclust in R; present and visualize the correlation between modules and features through the function labeledHeatmap; and finally, identify feature genes by evaluating the module membership (MM) and gene significance (GS) of genes.

### 2.5. Single-Sample Gene Set Enrichment Analysis (ssGSEA)

The metabolic activity of pathways was calculated using ssGSEA based on single-sample gene expression. KEGG metabolism pathways (https://github.com/wu-yc/scMetabolism/tree/main/data (accessed on 5 January 2025), which included 83 KEGG metabolism-related pathways [20], were analyzed for metabolic activity. ssGSEA was conducted using the gsva function in the “gsva” package (version 2.0.4). Finally, the correlations between the expression levels of hub genes and the metabolic activity of pathways were calculated.

### 2.6. Mouse Model of Heart Failure

The animal experiments received approval from the Animal Ethics Committee of Peking University First Hospital (J201713) and were carried out in compliance with the relevant animal ethical guidelines. Twelve healthy wild-type male C57BL/6 mice aged 6 to 8 weeks, were housed under specific pathogen-free (SPF) conditions with a 12-h light/dark cycle, controlled temperature (22 ± 2 °C). Mice were randomly assigned to control and Ang II groups (*n* = 6 per group) using a random number table. For the Ang II group, an osmotic pump (1.44 mg/kg/day) was subcutaneously implanted into the backs of the mice for 4 weeks to prepare heart failure model. At week 4, cardiac function was assessed using transthoracic echocardiography with the VEVO-2100 imaging system (VisualSonics, Toronto, ON, Canada). Mice were anesthetized with isoflurane and cardiac contractile function was evaluated through M-mode echocardiography. Blinded analysis of echocardiographic parameters (ejection fraction (EF) and fractional shortening (FS)) was performed by an independent researcher. Following the assessment, the mice were sacrificed to collect heart tissues.

### 2.7. Total RNA Extraction and RT-qPCR

Total RNA was extracted from mouse heart tissue samples with Trizol (15596018CN, Invitrogen, Carlsbad, CA, USA). A total of 2 μg of RNA was utilized for reverse transcription to synthesize cDNA. qPCR was performed using cDNA as the template and β-actin as the internal control, following the instructions provided with the Promega real-time PCR detection kit (A6002, Promega, Madison, CA, USA). The primer sequences are provided in Table 2.

### 2.8. Western Blot

A total of 10 mg of cardiac tissue was homogenized in RIPA lysis buffer (Beyotime, P0013B, Shanghai, China) supplemented with protease and phosphatase inhibitors (KangChen Bio-tech, KC-440, Beijing, China). The homogenate was centrifuged at 12,000×*g* for 10 min at 4 °C, and the supernatant was collected. The protein concentration was determined using a BCA assay kit (Beyotime, P0012, Shanghai, China). Then, 10 μg of total protein per sample was separated using 10% SDS-PAGE and transferred onto NC membranes (GE, 1060002, Boston, MA, USA). Membranes were blocked with 5% milk in PBST for 1 h at room temperature, followed by overnight incubation at 4 °C with primary antibodies: anti-SDC2 (1:2000, Proteintech, 67088, Wuhan, China) and anti-β-actin (1:5000, ZSGB-BIO, TA-09, Beijing, China). After washing, membranes were incubated with HRP-conjugated secondary antibodies (1:5000, Sigma, A0545/A4416, St. Louis, MO, USA) for 1 h at room temperature. Protein bands were visualized using an ECL kit (MeilunBio, MA0186-1, Dalian, China) and quantified using ImageJ software (version 1.53).

### 2.9. Receiver Operating Characteristic Analysis

Receiver operating characteristic (ROC) analysis is a statistical method for evaluating the accuracy of judgments. It combines sensitivity and specificity and presents the ability to correctly determine positive and negative samples at different cutoff values. The “pROC” package (version 1.18.5) was used to generate the ROC curve.

### 2.10. Identification of Differential Metabolites in Plasma Metabolomics

For the analysis of differential metabolites, the threshold criteria for statistical significance were adjusted *p*_value < 0.05 and Fold change > 1.2 or Fold change < 0.83. The expression data of differential metabolites were visualized in a volcano plot through the “ggplot2” package (version 3.5.1).

### 2.11. Drug Identification via DrugBank Database

The DrugBank database (https://go.drugbank.com/ (accessed on 19 January 2025)) is a leading resource for drug, drug–target, and pharmaceutical information [21]. We utilized the function for drug targets in the DrugBank database to explore potential drugs targeting the hub gene.

### 2.12. Statistical Analysis

All statistical analyses were conducted using R software (version 4.4.2). The Wilcoxon test or Student’s *t*-test was employed to assess the differences between two groups. Spearman’s correlation test was performed to evaluate the correlation between variables. For differential gene expression, metabolite screening, and pathway enrichment analyses, Benjamini–Hochberg false discovery rate (FDR) correction was applied, with a significance threshold of adjusted *p*_value < 0.05. For targeted validation experiments (qPCR and Western blot), statistical significance was assessed using raw *p*-values due to the limited number of comparisons. If data violated normality assumptions, non-parametric tests were applied. Data are presented as mean ± SD. *p* < 0.05 was considered statistically significant.

## 3. Results

### 3.1. Identification of DEGs in HF

**Figure 2:**
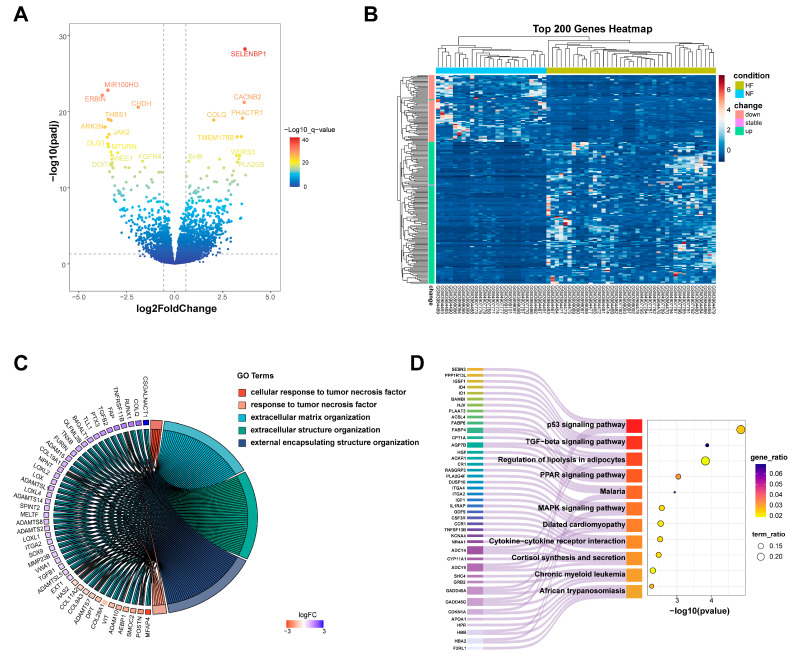
The identification of DEGs in HF. (**A**) A volcano plot showing significantly upregulated and downregulated genes in HF; (**B**) a heatmap showing the expression of the top 200 DEGs via hierarchical clustering; (**C**) a chord diagram showing the GO enrichment analysis results for DEGs in HF; (**D**) a Sankey diagram showing the pathway of KEGG enrichment analysis for DEGs in HF. DEGs: differentially expressed genes; GO: Gene Ontology; NF: non-failing; HF: heart failure; KEGG: Kyoto Encyclopedia of Genes and Genomes.

Three datasets (GSE161472, GSE133054, and GSE135055), including 26 HF samples and 40 normal tissues were integrated, and batch effects were removed by ComBat (via the “sva” R package, version 3.54.0). Normalized expression levels of the integrated samples are presented in Figure S1, revealing a largely similar distribution of gene expression across samples from all datasets. This ensures that the integrated dataset is suitable for identifying DEGs. In comparison to the control group, a total of 1115 DEGs were found with the criteria Fold change > 1.5 or Fold change < 0.67 and adjusted *p*_value < 0.05, with 701 upregulated genes and 414 downregulated genes (Figure 2A). The top 200 DEGs in each group are visualized in Figure 2B.

GO and KEGG enrichment analyses were conducted on all differential genes to identify their biological functions and the signaling pathways related to the development of HF. The GO enrichment analysis revealed that the DEGs were primarily enriched in biological processes, including cellular response to tumor necrosis factor and extracellular matrix organization (Figure 2C). The results of KEGG enrichment analysis demonstrated that the DEGs are significantly involved in important signaling pathways, such as the p53 signaling pathway, TGF-β signaling pathway, and regulation of lipolysis in adipocytes (Figure 2D). In summary, the enrichment analysis results indicate that the mechanisms of HF involve a complex regulatory network, including alterations in cell cycle regulation (such as the p53 signaling pathway), disruptions in lipid metabolism (such as the PPAR signaling pathway and the regulation of lipid metabolism in adipocytes), and the dysregulation of inflammatory responses (such as cytokine–cytokine receptor interactions and the TGF-β signaling pathway). Moreover, pathways associated with cellular stress responses (such as the MAPK signaling pathway) and calcium homeostasis (such as the calcium signaling pathway) were also significantly enriched, indicating that these processes may be pivotal in disease progression and its underlying molecular mechanisms.

### 3.2. Identification of Metabolism-Related Hub Genes in HF

**Figure 3:**
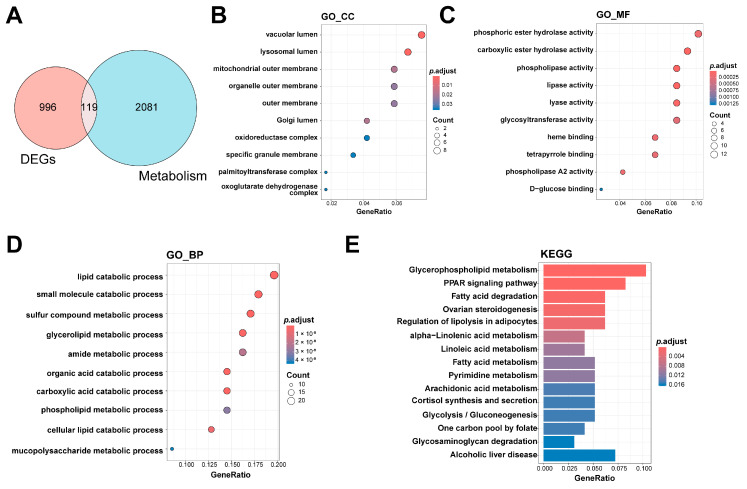
Identification and enrichment analysis of metabolism-related hub genes in HF. (**A**) The Venn diagram shows the overlap of DEGs (red circle) and metabolism-related genes (blue circle); (**B**) enrichment results for the CC category in GO analysis; (**C**) enrichment results for the MF category in GO analysis; (**D**) enrichment results for the BP category in GO analysis; (**E**) the results of enrichment analysis of KEGG pathways. CC: cellular component, DEGs: differentially expressed genes, GO: Gene Ontology, HF: heart failure; KEGG: Kyoto Encyclopedia of Genes and Genomes; MF: molecular function; BP: biological process.

After intersecting 2200 metabolism-related genes with the 1115 differential genes, 119 metabolism-related DEGs were identified (Figure 3A). The cellular component (GO_CC) analysis revealed that the intersected genes are enriched in organelle-related locations including the vacuolar lumen, lysosomal lumen, and mitochondrial outer membrane (Figure 3B), indicating that these genes may be closely associated with energy metabolism and substance transport processes. The molecular function (GO_MF) analysis indicated that the intersected genes are primarily involved in functions including phosphoric ester hydrolase activity, carboxylic ester hydrolase activity, and phospholipase activity (Figure 3C), indicating that they may be crucial in metabolic processes via enzymatic reactions. The biological process (GO_BP) enrichment analysis revealed that the intersected genes are significantly involved in metabolic processes such as the lipid catabolic process, small molecule catabolic process, and glycolipid metabolic process (Figure 3D), further supporting their key roles in metabolic regulation.

The findings of the KEGG pathway enrichment analysis further implied that the metabolism-related DEGs are involved in a complex regulatory network, including disruptions in lipid metabolism (such as glycerophospholipid metabolism and fatty acid metabolism), changes in energy homeostasis (such as glycolysis/gluconeogenesis and fatty acid degradation), and abnormalities in signal transduction regulation (such as the PPAR signaling pathway and the regulation of lipolysis in adipocytes). In addition, pathways associated with inflammation regulation (e.g., arachidonic acid metabolism) and hormone synthesis (e.g., steroidogenesis regulation) were significantly enriched. These results indicate that these metabolic pathways may be crucial in the progression of HF.

### 3.3. Screening of Metabolism-Related Hub Genes Associated with HF via Machine Learning

Next, we employed three machine learning algorithms—Lasso, Random Forest, and XGBoost—to further select hub genes from the 119 metabolism-related DEGs.

**Figure 4:**
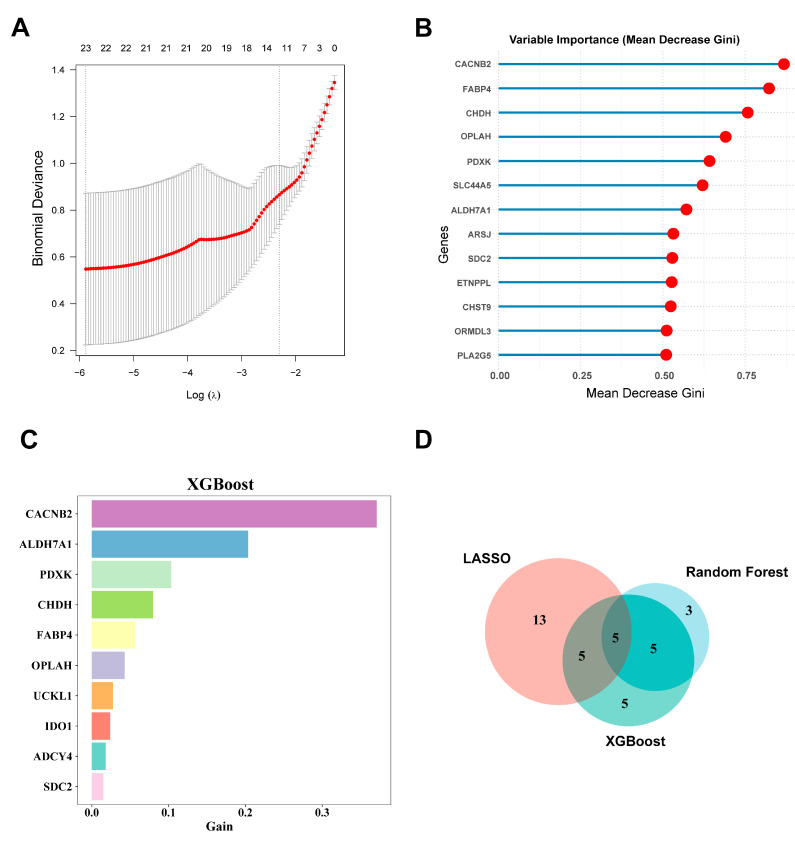
Screening of hub genes using machine learning. (**A**) Key feature genes were screened by performing LASSO regression analysis; (**B**) the top 10 most important genes identified by Random Forest based on Mean Decrease Gini; (**C**) genes ranked by importance based on their Gain in the XGBoost method; (**D**) a Venn diagram illustrating the overlap of important genes identified through the three machine learning methods. LASSO: least absolute shrinkage and selection operator; XGBoost: Extreme Gradient Boosting.

In the LASSO regression analysis, 10-fold cross-validation was applied to obtain the optimal model, with the parameter family set to binomial. Finally, a gene coefficient plot (Figure S2A) and a binomial deviation plot were generated (Figure 4A). The hub genes were identified from the model according to the corresponding parameters. In the end, LASSO regression identified 23 core genes: *CPT1A*, *ADHFE1*, *HGSNAT*, *ADCY4*, *SLC44A5*, *FABP4*, *PLCH1*, *FUT1*, *ACOT11*, *GNMT*, *HEXA*, *HK3*, *HMGCS2*, *APOA1*, *PLA2G2A*, *UCKL1*, *CYP26B1*, *SDC2*, *LPIN3*, *CACNB2*, *CHAC1*, *CHST9*, and *ABCG2*.

The Random Forest algorithm visualized the model’s diagnostic errors and the importance ranking of gene variables. The diagnostic error in the Random Forest model is visualized in Figure S2B. The candidate hub genes are ranked according to their importance metric (Mean Decrease Gini) (Figure 4B). Thirteen hub genes with Mean Decrease Gini > 0.5 were selected: *CACNB2*, *FABP4*, *CHDH*, *OPLAH*, *PDXK*, *SLC4A5*, *ALDH7A1*, *ARSJ*, *SDC2*, *ETNPPL*, *CHST9*, *ORMDL3*, and *PLA2G5*.

After creating a decision tree model, the XGBoost machine learning algorithm directly provides the importance score for each feature. By calculating and ranking the importance scores of each feature in the dataset (Figure 4C), we identified 20 hub genes: *CACNB2*, *ALDH7A1*, *PDXK*, *CHDH*, *FABP4*, *OPLAH*, *UCKL1*, *IDO1*, *ADCY4*, *SDC2*, *CHST9*, *PPT1*, *SREBF1*, *PLA2G5*, *GNMT*, *SLC44A5*, *GPC3*, *PLCH1*, *CHAC1*, and *CYB5B*. Finally, we obtained five hub genes that were identified by all three machine learning methods: *CACNB2*, *CHST9*, *FABP4*, *SDC2*, and *SLC44A5* (Figure 4D).

### 3.4. Identification of Gene Modules in HF Using WGCNA

**Figure 5:**
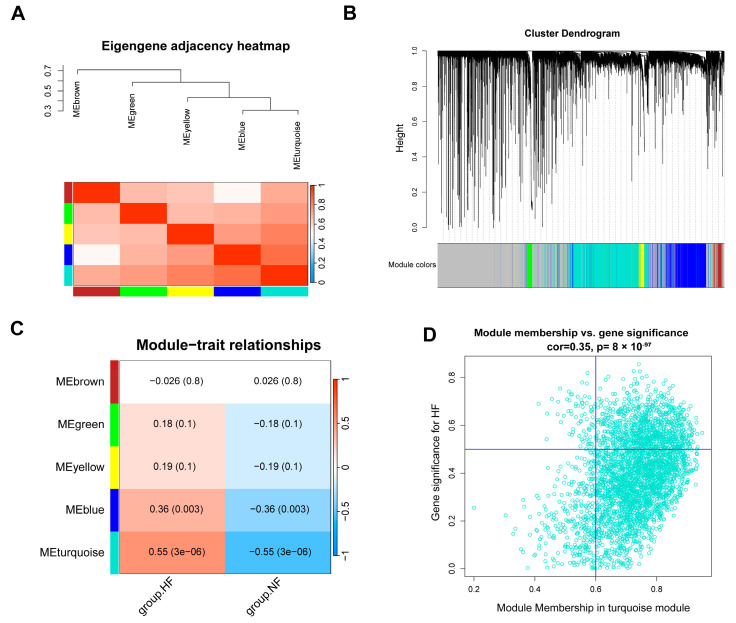
The identification of gene modules in HF using WGCNA. (**A**) A similarity heatmap of co-expression modules of feature genes; (**B**) a gene module hierarchical clustering tree; (**C**) a module–trait relationship heatmap showing the correlation between different modules and traits; (**D**) the relationship between MM and GS in the turquoise module. WGCNA: Weighted Gene Co-expression Network Analysis; MM: module membership; GS: gene significance; HF: heart failure; NF: non-failing.

To identify the gene modules associated with HF, we obtained another dataset with a sufficient number of samples. After preprocessing and normalizing the RNA-seq dataset GSE116250, a total of 64 samples and 12,437 genes were obtained. A sample clustering tree was used to detect outlier samples and show the corresponding grouping information (Figure S3A). The appropriate soft threshold β, was determined to be power = 16 (R^2^ > 0.85) for constructing the WGCNA network (Figure S3B). Using TOM (topological overlap matrix), a hierarchical clustering tree of genes was constructed, and the dynamic tree-cut method was employed to divide the genes into different color modules. Finally, five gene modules were identified (Figure 5A,B). Among them, the turquoise module (3339 genes, *p* = 3 × 10^−6^, r = 0.55) was the most positively correlated with the heart failure phenotype (Figure 5C). With the filtering criteria of gene significance (GS) > 0.5 and gene module correlation (MM) > 0.6, 925 genes from the turquoise module were selected as core genes (Figure 5D).

### 3.5. SDC2 Is a Key Gene Associated with HF

**Figure 6:**
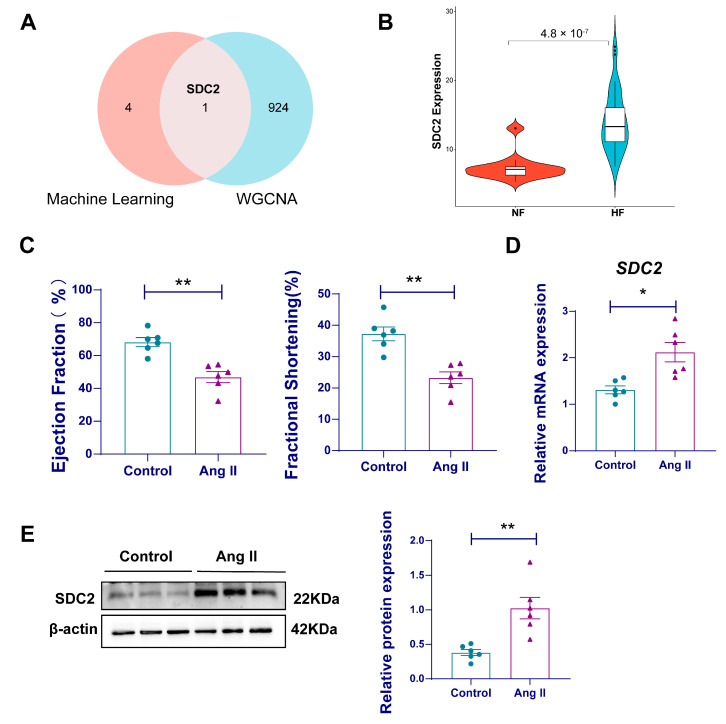
*SDC2* is a key gene associated with HF. (**A**) A Venn diagram showing the overlap of important genes selected using machine learning and WGCNA; (**B**) a violin plot of *SDC2* gene mRNA expression; (**C**) cardiac function of AngII-induced HF mouse (*n* = 6 per group); (**D**) *SDC2* mRNA expression was experimentally detected in the myocardial tissue of a mouse HF model using RT-qPCR (*n* = 6 per group); (**E**) SDC2 protein expression was experimentally detected in the myocardial tissue of a mouse HF model using a western blot (*n* = 6 per group). Data are presented as mean ± SD. HF: heart failure; NF: non-failing; RT-qPCR: reverse transcription quantitative PCR; *SDC2*: Syndecan 2; WGCNA: Weighted Gene Co-expression Network Analysis. * *p* < 0.05, ** *p* < 0.01.

Next, we validated the hub genes obtained using machine learning in the WGCNA. Syndecan-2 (*SDC2*) was the only gene identified as a key feature gene in both methods (Figure 6A). The gene expression of *SDC2* was also validated using the GSE116520 dataset, and the results showed that *SDC2* expression was considerably elevated in the HF group when compared with controls (Figure 6B). To further validate our findings, we also constructed an Ang II-induced HF mouse model. The echocardiography results showed significant impaired cardiac function in the mice of the Ang II group, with decreased ejection fraction and fractional shortening compared to controls (Figure 6C). RT-qPCR revealed that *SDC2* mRNA expression was markedly elevated in the cardiac tissues of HF mice (Figure 6D), consistent with the findings from bioinformatics analysis. This transcriptional upregulation was further proved at the protein level using a Western blot, which showed a significant elevation of SDC2 in HF mice (Figure 6E).

### 3.6. SDC2 Expression Is Associated with Metabolic Changes in HF

**Figure 7:**
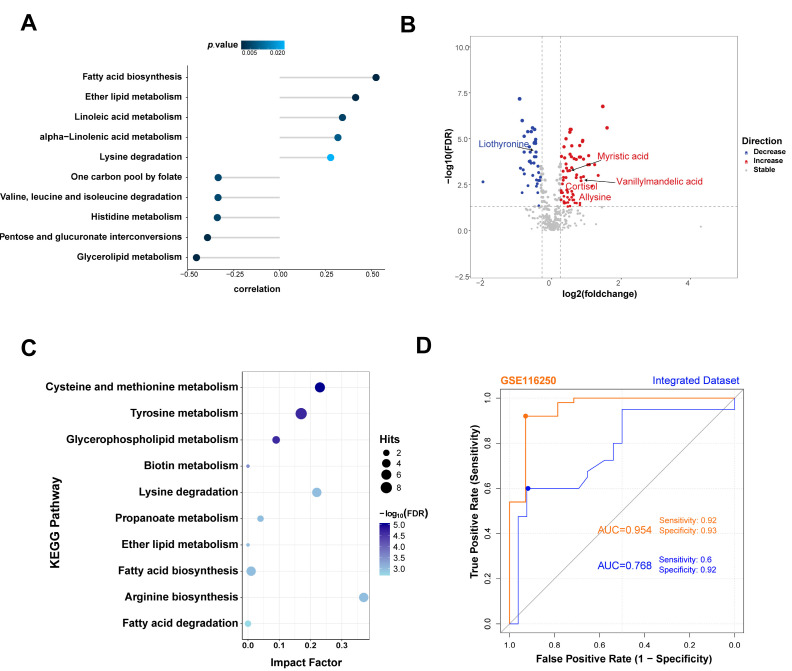
The association between metabolic changes and *SDC2* expression in HF. (**A**) Correlation analysis of *SDC2* expression with metabolism pathways. (**B**) Differential metabolites in the HF group compared with the NF group. (**C**) KEGG enrichment analysis of differential metabolites between HF and NF; (**D**) ROC curves of *SDC2* in the GSE116250 and integrated dataset showing the HF diagnostic performance. HF: heart failure; KEGG: Kyoto Encyclopedia of Genes and Genomes; NF: non-failing; ROC: receiver operating characteristic; *SDC2*: Syndecan 2.

The correlation between the *SDC2* expression level and the metabolic activity of pathways was also analyzed. The results indicated that *SDC2* expression was positively correlated with fatty acid biosynthesis, ether lipid metabolism, and others and significantly negatively correlated with glycerolipid metabolism, pentose and glucuronate interconversions, etc. (Figure 7A). We further validated the results using published plasma metabolomics data from HF patients [22]. The results showed that myristic acid in fatty acid biosynthesis, cortisol in steroid hormone biosynthesis, allysine in lysine degradation, vanillylmandelic acid, and liothyronine in tyrosine metabolism were significantly changed in the plasma of HF patients compared with controls and regarded as biomarkers of HF (Figure 7B). Pathway enrichment analyses of all the differential metabolites showed the same pathways related to the expression of *SDC2* (Figure 7C).

### 3.7. Potential Clinical Applications of SDC2 in the Diagnosis and Treatment of HF

To validate the diagnostic capability of *SDC2* in HF, we performed an ROC analysis on both the GSE116250 and integrated datasets. The ROC curves showed that *SDC2* had ideal diagnostic performance: in the GSE116250 dataset, the ROC curve showed an AUC value of 0.954, with a sensitivity of 0.92 and a specificity of 0.93; in the integrated dataset, the AUC value was 0.768, with a sensitivity of 0.6 and a specificity of 0.92 (Figure 7D).

In addition, we searched the DrugBank database to identify potential drugs targeting SDC2. The results showed two drugs targeting SDC2: Palifermin, which is used for the prevention and treatment of oral mucositis, and Efanesoctocog alfa, which is indicated for the treatment of hemophilia A. However, to date, no drugs targeting SDC2 for the treatment of HF have been developed.

## 4. Discussion

To screen the hub genes related to metabolism in heart failure, we first screened differentially expressed genes linked to heart failure in integrated datasets derived from human heart tissue samples. A total of 701 upregulated and 414 downregulated genes were obtained. The enrichment analysis of the 1115 genes revealed that they mainly affect the cell cycle, lipid metabolism, inflammatory response, cellular stress response, and calcium homeostasis. Then, by intersecting differentially expressed genes and metabolism-related genes, a set of 119 metabolism-related genes potentially linked to heart failure was identified. These genes may serve as a bridge between metabolic abnormalities and disease progression. To identify genes linked to high-importance traits from these 119 genes, we applied three widely used machine learning algorithms for further validation. LASSO regression identified 23 important genes by controlling the regularization parameter. Random Forest selected 13 important genes based on their Mean Decrease Gini scores. XGBoost (version 1.7.8.1) identified 20 important genes by evaluating the Gain value of each gene. A total of five genes were validated by all methods.

Next, to validate the results obtained from the combined dataset, we downloaded another heart failure transcriptome dataset and performed WGCNA to identify modules and genes highly correlated with the HF phenotype. Genes with similar expression patterns were grouped into different modules. The results showed that the turquoise module was highly correlated with the HF phenotype. With filtering criteria of GS > 0.5 and MM > 0.6, 925 core genes were ultimately identified. Among the 925 core genes, only *SDC2* from the five previously identified genes appeared in the core gene set. The *SDC2* gene was repeatedly confirmed in both machine learning and WGCNA screenings.

SDC2, a transmembrane (type I) heparan sulfate proteoglycan, also known as fibroglycan, was initially biochemically characterized as a major cell surface transmembrane protein that expresses heparan sulfate glycosaminoglycans in lung fibroblasts [23,24]. The SDC2 protein primarily consists of three regions: the N-terminal extracellular domain, the single-transmembrane domain, and the cytoplasmic tail domain [13]. The transmembrane localization of SDC2 enables it to function as both a receptor and a co-receptor, participating in various cellular functions, including cytoskeletal rearrangement, intercellular communication, cell adhesion, and cell migration [25]. SDC2 also plays a crucial role in the development and pathogenesis of several diseases. During embryogenesis, SDC2 is crucial for the formation of the nervous system and angiogenesis [26,27]. However, SDC2 has also been reported to interact with amyloid-β peptides, leading to their accumulation on the cell surface and the subsequent formation of amyloid plaques. This process is associated with the development of neurodegenerative diseases, such as Alzheimer’s disease [28]. Moreover, recent studies have found that SDC2 stimulates the migration of cancer cells during the development of intestinal tumors, and its methylation level has shown high diagnostic value and prognostic significance in intestinal tumors [29,30]. An integrated bioinformatics analysis was performed to investigate the function of heparan sulfate in dilated cardiomyopathy, which revealed that SDC2 was strongly correlated with collagen I and collagen III in the cardiac fibroblasts of hearts with dilated cardiomyopathy [31]. However, the function of SDC2 in the progression of HF remains unclear.

*SDC2* expression was significantly upregulated in HF patients in the GSE116520 dataset. The mRNA and protein expression of SDC2 was further verified in Ang II-induced mice, which showed cardiac systolic dysfunction. Our analysis also revealed a strong association between *SDC2* expression and metabolic alterations, particularly in fatty acid biosynthesis, ether lipid metabolism, and glycerolipid metabolism. These findings suggest that SDC2 may be a critical mediator of metabolic shifts in heart failure. In addition, SDC2 might be a key target in the pathogenesis of HF, and its upregulation might, to some extent, contribute to its onset. Regarding the clinical significance of *SDC2* in the diagnosis and treatment of HF, the ROC curves showed the capability of *SDC2* to differentiate HF patients from non-HF individuals, highlighting its potential as a non-invasive diagnostic biomarker. Moreover, the exploration of the Drugbank database showed that there were two possible drugs related to SDC2. However, although SDC2 is implicated in several medical conditions, no drugs have yet been developed to specifically target SDC2 in the context of HF treatment. This opens up an exciting avenue for future research, where SDC2 could be explored as a novel therapeutic target, particularly given its involvement in the metabolic pathways that contribute to heart failure pathogenesis.

High-sensitivity troponin T (hs-TnT) and N-terminal pro B-type natriuretic peptide (NT-proBNP) have been widely recognized as essential biomarkers for the diagnosis, prognosis, and management of heart failure [32]. NT-proBNP reflects myocardial wall stress and is frequently used to assess HF severity [33,34]. Additionally, hs-TnT serves as a marker of myocardial injury and has strong prognostic value in both acute and chronic HF settings [35,36]. While NT-proBNP and hs-TnT remain the gold standard biomarkers, SDC2 may offer complementary value by capturing distinct pathological processes that are not fully represented by these traditional markers. Future studies are warranted to validate the clinical applicability of SDC2 and to determine its potential for integration into current HF diagnostic and prognostic models.

However, this study has certain limitations. First, although *SDC2* was identified as a hub gene in the progression of heart failure through comprehensive transcriptome analysis and its mRNA expression was experimentally validated in an HF mouse model, more experiments are required to clarify the specific function of SDC2 in the pathogenesis of HF and metabolism. Second, *SDC2* gene expression is important, but also the presence of sequence-altering mutations is a potentially important indicator of causal association with heart failure. Future studies are warranted to explore the potential contribution of *SDC2* mutations to metabolic imbalances or heart failure. Third, the sample size may restrict the generalizability of the findings. Consequently, subsequent research should enhance the experimental validity and increase the sample size to reinforce and broaden the findings of this study.

## 5. Conclusions

In conclusion, we systematically explored the gene expression characteristics in heart failure by integrating multiple bioinformatics analysis methods, including differential expression analysis, machine learning algorithms, WGCNA, and metabolic changes. In addition, we identified *SDC2* as a potential key gene involved in heart failure. Future functional studies on SDC2 may provide novel targets for the diagnosis and treatment of HF.

## Figures and Tables

**Table 1:** Detailed information on the 4 datasets.

Datasets	Year	Sample Size	Platform	Read Length	Layout	Sequencing Technology	Instrument Model
GSE161472 [12]	2020	9 NF; 12 HF	GPL11154	150 bp	Paired	Illumina	HiSeq 2000
GSE133054 [13]	2019	8 NF; 7 HF	GPL18537	70 bp	Paired	Illumina	NextSeq 500
GSE135055 [14]	2020	9 NF; 21 HF	GPL16791	150 bp	Paired	Illumina	Hiseq 2500
GSE116250 [15]	2018	14 NF; 50 HF	GPL16791	50 bp	Single	Illumina	Hiseq 2500

NF: non-failing; HF: heart failure.

**Table 2:** Primer sequences.

Primer	Sequence
SDC2-Forward	ACAGAAGTTCTAGCAGCCGTC
SDC2-Reverse	TGGATGGTTTGCGTTCTCCA
β-actin-Forward	CACTGTCGAGTCGCGTCC
β-actin-Reverse	TCATCCATGGCGAACTGGTG

## Data Availability

Raw gene expression profiles of GSE161472, GSE133054, GSE135055 and GSE116250 can be downloaded from the GEO database.

## Supplementary Materials

The following supporting information can be downloaded at: https://www.mdpi.com/article/10.3390/genes16030305/s1, Figure S1: Data integration. Figure S2: Screening of hub genes via machine learning. Figure S3: Identification Gene Modules in HF using WGCNA.

## Abbreviations

The following abbreviations are used in this manuscript:

BP	Biological process
CC	Cellular component
DEGs	Differentially expressed genes
GO	Gene Ontology
GS	Gene significance
HF	Heart failure
KEGG	Kyoto Encyclopedia of Genes and Genomes
LASSO	Least absolute shrinkage and selection operator
MF	Molecular function
MM	Module membership
NF	Non-failing
RT-qPCR	Reverse transcription quantitative PCR
SDC2	Syndecan 2
WGCNA	Weighted Gene Co-expression Network Analysis
XGBoost	Extreme Gradient Boosting
