# Supplementary material for: Identification of Metabolism-Related Hub Genes in Heart Failure via Comprehensive Transcriptome Analysis
Source: Genes (Basel). 2025 Mar 3;16(3):305. doi: 10.3390/genes16030305 (PMC11941980; doi:10.3390/genes16030305)
Supplement: Supplementary file 1 [file genes-16-00305-s001.zip › genes-3483348-supplementary.pdf]

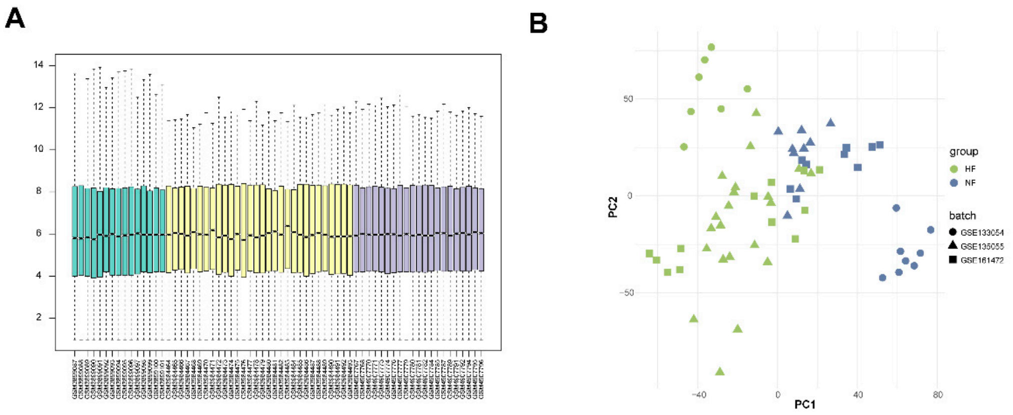

Supplementary Figure 1. Data integration. (A) Boxplot showing the standardized distribution of expression levels of different samples after integration; (B) Principal component analysis (PCA) diagram shows the clustering of samples according to gene expression profile.

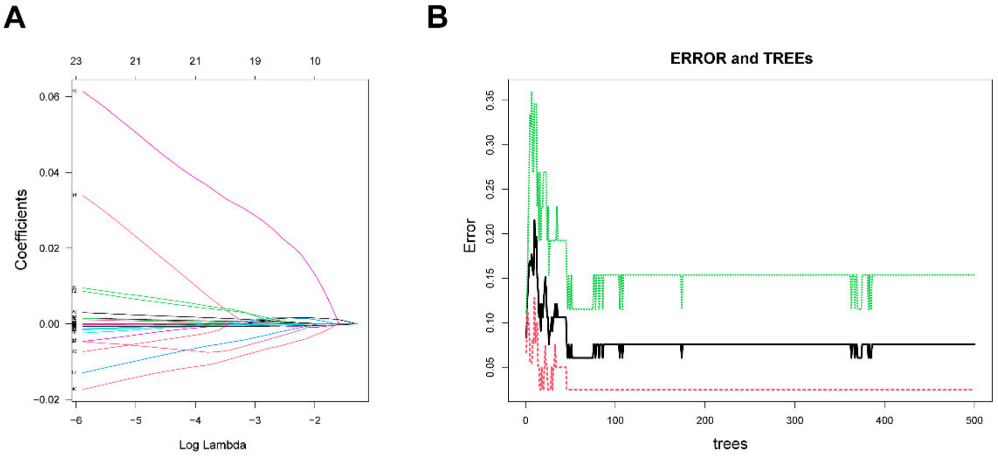

Supplementary Figure 2. Screening of hub genes via machine learning. (A) Gene coefficient plot in LASSO regression analysis; (B) Visualization of diagnostic error in the Random Forest model.

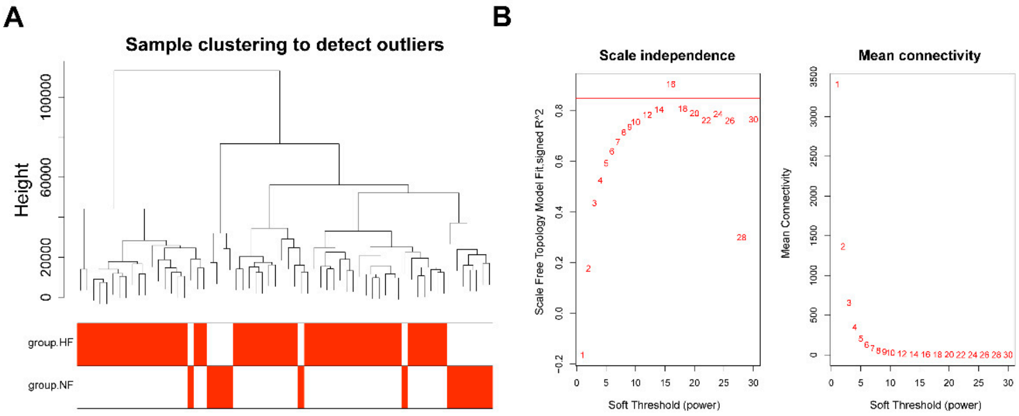

Supplementary Figure 3. Identification Gene Modules in HF using WGCNA. (A) Sample clustering tree used to detect outlier samples and display the corresponding grouping information; (B) Selection of the optimal soft threshold based on the scale-free network fit index and mean connectivity.
